# Is deeper always better? Evaluating deep learning models for yield forecasting with small data

**DOI:** 10.1007/s10661-023-11609-8

**Published:** 2023-09-06

**Authors:** Filip Sabo, Michele Meroni, François Waldner, Felix Rembold

**Affiliations:** European Commission, Joint Research Centre, Ispra, Italy

**Keywords:** Convolutional neural networks, Agriculture, Remote sensing, Food security

## Abstract

Predicting crop yields, and especially anomalously low yields, is of special importance for food insecure countries. In this study, we investigate a flexible deep learning approach to forecast crop yield at the provincial administrative level based on deep 1D and 2D convolutional neural networks using limited data. This approach meets the operational requirements—public and global records of satellite data in an application ready format with near real time updates—and can be transferred to any country with reliable yield statistics. Three-dimensional histograms of normalized difference vegetation index (NDVI) and climate data are used as input to the 2D model, while simple administrative-level time series averages of NDVI and climate data to the 1D model. The best model architecture is automatically identified during efficient and extensive hyperparameter optimization. To demonstrate the relevance of this approach, we hindcast (2002–2018) the yields of Algeria’s three main crops (barley, durum and soft wheat) and contrast the model’s performance with machine learning algorithms and conventional benchmark models used in a previous study. Simple benchmarks such as peak NDVI remained challenging to outperform while machine learning models were superior to deep learning models for all forecasting months and all tested crops. We attribute the poor performance of deep learning to the small size of the dataset available.

## Introduction

Crop yield forecasting is essential for planning agricultural production and ensuring the safety of a nation’s food supplies and is also of value to many stakeholders, including farmers, agronomists and policymakers. A key challenge of addressing food security is how to obtain reliable yield forecasts with sufficient lead time, especially in food-insecure regions where the crop performances can be strongly influenced by climate variability (Baffour-Ata et al., 2021). With increasing impact of weather variability and extremes on food security (Hasegawa et al., 2021), governments need to anticipate crop production losses to respond appropriately. More timely and accurate forecasts would help support related crucial policy decisions.

Multitemporal optical remote sensing and meteorological data are appropriated data sources for yield forecasting. With the objectives of increasing automation, standardizing the yield forecasting process, and boosting accuracy and timeliness, machine learning (ML) and deep learning (DL) approaches are increasingly being employed to process Earth Observation data (van Klompenburg et al., 2020). DL is particularly appealing due to its ability to model complex, highly nonlinear, relationships between yield and biophysical or meteorological variables. Furthermore, DL models do not require feature engineering, that is, they can automatically discover the relationships between the input data and yield by extracting relevant features from the input data. A variety of DL architectures have been used for crop yield forecasting including (1) one-dimensional convolutional neural networks (1D CNN), where the kernel slides along one direction of the input time series data (Wolanin et al., 2020); (2) two dimensional convolutional neural networks (2D CNN), where the input data is transformed into fixed-bins histograms with multiple bands (Sun et al., 2020; You et al., 2017); (3) long short-term memory (LSTM) (Ju et al., 2021) tailored for processing sequential data and (4) autoencoders, an unsupervised DL technique (Ma et al., 2019). Models combining CNN and LSTM were also proposed (Sun et al., 2020), to leverage both spatial (CNN) and temporal input features (LSTM). Several studies (Schwalbert et al., 2020; Cao et al., 2021; Srivastava et al., 2022) have reported that DL models have performed better than various conventional ML methods (e.g. Lasso, Support Vector Regressor, Random Forest, XGBoost). On the other hand, 2D CNNs and LSTMs have been reported as not advantageous for crop yield forecast when compared to XGBoost, especially for a small feature datasets (Kang et al., 2020).

In this study, we investigated whether a flexible DL approach could circumvent the shortcoming due to working with small data and outperform conventional and machine learning benchmarks. We focus on the provincial level yield forecasting using 1D and 2D convolutional neural networks. The best model architecture is identified during efficient and extensive hyperparameter optimization, when unpromising trials are discarded early based on a greedy cross-validation approach. To demonstrate the relevance of our approach, we hindcast (2002–2018) the yields of Algeria’s three main crops (barley, durum and soft wheat) and contrast its performance with ML algorithms and conventional benchmark. Our approach uses free and open near real-time predictors from the European Commission Joint Research Centre - Anomaly hotSpots of Agricultural Production (https://mars.jrc.ec.europa.eu/asap/, Rembold et al., 2019) and meets the operational requirements of the application and is transferrable to any country where a cropland mask and reliable yield statistics are available.

## Data and methods

### Study area and yield data

**Fig. 1:**
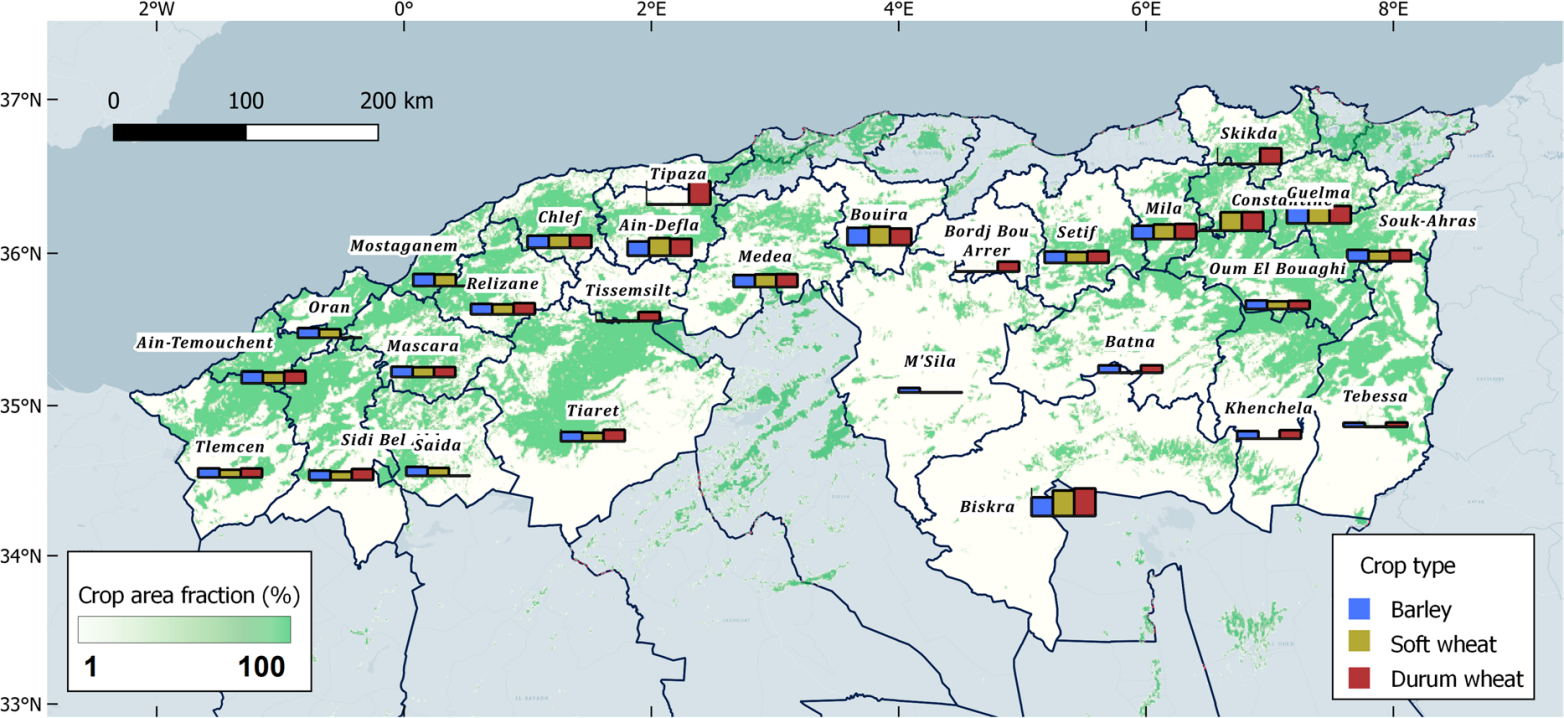
Study area in Algeria shown with percent crop area and mean yield per province. We selected the most productive provinces up to 90% national production; therefore, one province may be used for one cereal and not for the other. We report average yield in provinces that were used in the study. Crop percent area refers to the percentage of crop in the 1-km grid of the ASAP system

The study area was located in Algeria, a cereal producer facing high inter-annual climatic variability (Benmehaia et al., 2020). Our analysis focussed on more than 20 provinces (Fig. 1) representing 90% of the national mean crop production for the main cereal crops: durum wheat, barley and soft wheat (Fig. 1). The climate ranges from semi-arid in the South to Mediterranean in the North. Cereals are generally rainfed (only 3% of the cereal area is irrigated according to FAOSTAT). The crop calendar extends from sowing, between October and November depending on autumn rainfall, and harvest from May to July. Crop yields ranged from 0.5 to 3 t/ha.

Official yield statistics were provided by the Direction des Statistiques Agricoles et des Systèmes d’Information - Ministère de l’Agriculture et du Développement Rural (DSASI-MADR). The time range of the analysis (from 2001/2002 to 2017/2018 crop seasons) was determined by the availability of both MODIS imagery used in this study (from 2001) and wheat yield official statistics (up to 2018 harvest). As a result, the total number of yield data points (*n* = 17 years × no. provinces) was 408, 391 and 340 for durum wheat, barley and soft wheat, respectively. Our experimental context can be thus defined as data poor. More details about the study area characteristics and yield data statistics can be found in Meroni et al. (2021).

### Remotely sensed and categorical data

We predicted provincial yields using time series of four explanatory variables at 10-day time interval: NDVI from MODIS, air temperature and global radiation from ERA5 ECMWF and rainfall estimates from CHIRPS (Table 1). NDVI and climate data were downloaded from the Joint Research Centre (JRC) early warning system, Anomaly hotSpots of Agricultural Production (ASAP) (https://mars.jrc.ec.europa.eu/asap/download.php) (Rembold et al., 2019) as tabular data - aggregated in time (10 days) and in space (GAUL1 Administrative Unit) using the ASAP cropland mask. The cropland mask is an area fraction image at 1-km spatial resolution, expressing the percentage of the pixel that is covered by cropland. Aggregation in space is thus a weighted average of all province pixels using the area fraction as weighting factor.

**Table 1:** Input data used in this study

Category	Variable	Spatial resolution	Source
Satellite observation of vegetation	NDVI	1 km	MODIS MOD13A2 and MYD13A2 V006 (Meroni et al., 2019)
Meteorological data	Precipitation	5 km	CHIRPS 2.0 (Funk et al., 2015)
	Temperature	25 km	Elaboration on ECMWF ERA5 data (European Commission. Joint Research Centre., 2017)
	Global radiation		
Tabular data	Administrative code	Regional	Anomaly hotSpots of Agricultural Production (ASAP) (Rembold et al., 2019)

In addition to the NDVI and climate data, we used the province code from ASAP as an additional, categorical, input to the model as it improved the prediction capacity of the ML models (Meroni et al., 2021). This information could help the model in discriminating unobserved different management practices or soil properties among regions. These categorical variables were transformed into numerical features (one-hot encoding, i.e. replacing the code by new binary variables, one per unique categorical value) and passed as an additional input to DL models. The input data were normalized using min-max scaling before ingesting them into the deep learning workflows.

### Deep learning models

We evaluated two types of DL architectures: 1D and 2D CNNs. The architecture of both types of network were flexibly defined and optimized based on their hyperparameters (parameters that are defined in order to control the learning process (Table 2)):

- The 1D kernel size is the sliding window length;
- The 2D kernel size is the kernel matrix size in terms of width and height;
- The stride represents the sliding step used in convolutional operations;
- Pooling is used to replace each patch (1D or 2D) with a single output using mean or maximum operation. Spatial pyramid pooling is a pooling that removes the constrain of fixed size input by applying several levels of max pooling to the input image;
- Dense or fully connected layer is a transformation (linear in this study) in which every input is connected to every output from the previous layer;
- Number of epochs was in the range 100–250 with a step of 50.
- Tested batch size values were 64 and 128 with learning rate values of 0.001, 0.01 and 0.1.

**Table 2:** 1D and 2D CNN tested hyperparameters with their range of possible values in this experiment

Layer	Hyperparameter	Range
Convolution layers	Number of convolutional filters 1D Number of convolutional filters 2D	From 5 to 15 by step of 5 From 10 to 30 by step of 5
	Dropout rate Kernel size 1D	0.001 or 0.01 2 or 3
	Kernel size 2D Stride	From 3 to 9 by step of 3 2 or 3
Average pooling layer (1D) spatial pyramid pooling (2D)	Pooling size (1D) Pyramid bins (2D)	2 or 3 2 or 3
Regression head	Number of dense layers	0 or 1
	Number of units in the dense layer	0 or 16
Tabular inputs	Activation function	ReLU or sigmoid
	Number dense layers	0 or 1
	Number of units in the dense layer	From 10 to 30 by step of 5

#### 1D CNN

**Fig. 2:**
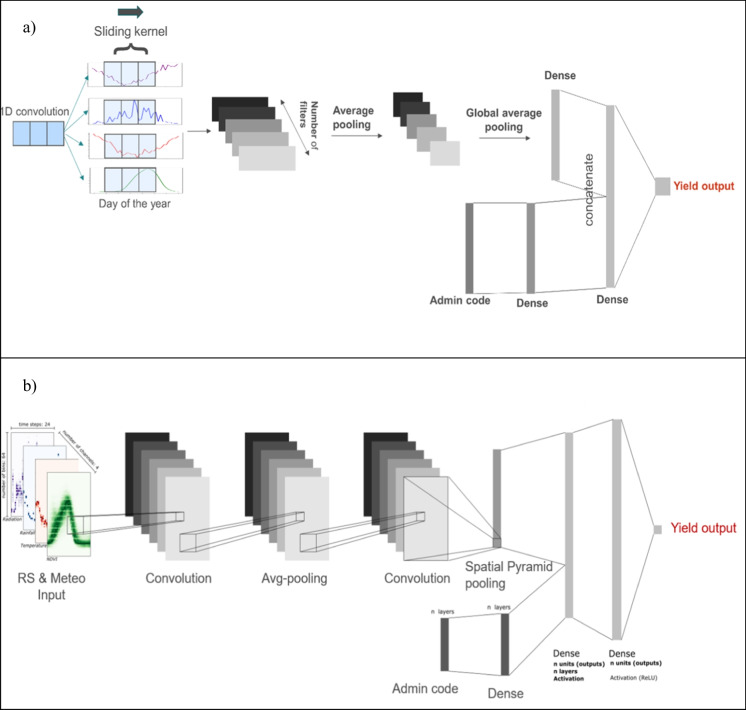
Examples of a 1D and a 2D convolutional neural network. **a** The 1D CNN. **b** The 2D CNN. In both cases, the upper branch of the network extracts features from the input data, while the lower branch extracts feature from the administrative code

For 1D CNNs, kernels slide along one dimension, i.e. the temporal dimension of the input time series (Fig. 2a). The models were provided with average time series extracted at the provincial level for cropland area. A series of 1D convolutional filters were first applied to the 4-channel input time series followed by an average pooling layer. After the second convolutional layer, the data were passed to the global average pooling layer which averaged the inputs along the time dimension for each channel. One hot encoded province IDs (administrative code) acted as a second, independent, input. They were first passed through a fully connected (dense) layer and then concatenated with the outputs from the flattened global average pooling layer. Finally, a dense layer with linear activation function was used to predict the final yield.

#### 2D CNN

**Fig. 3:**
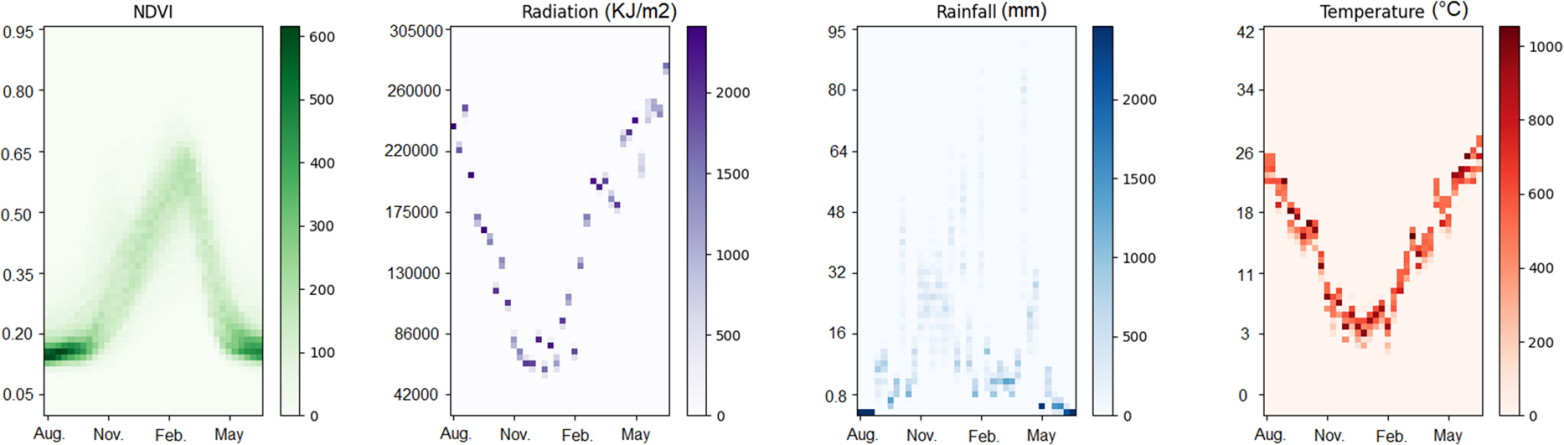
Histogram representation of NDVI and climate data for Ain-Defla province in 2006. The x-axis corresponds to the time step (10 days) counting from August, and the y-axis to the physical variable value. Density of observations is colour coded with the variable-specific colour bars

We treated raw images as histograms of pixel counts, which helps avoid overfitting and alleviates the scarcity of data (You et al., 2017). In doing so, pixel values within a cropland map were discretized for each input variable into 64 bins and obtain a histogram representation (Fig. 3). For each pixel within the cropland mask, the pixel count intensity corresponded to the cropland proportion of that pixel. By obtaining the histogram representations of the sequence of multi-band images and concatenate them through time dimension, one can produce compact histogram representations of the sequence of images taken along the growing season. As such, these 3D histograms capture both the temporal component of the data and their spatial information.

While the average value used in 1D CNNs compressed all the spatial information into one single value per administrative unit, the histogram maintains the information about the distribution. For example, let us consider a province where the crop status is equally distributed in a 50% high NDVI and a 50% low NDVI. The signal, in the 1D CNN setup, will be represented by average conditions. The histogram instead will preserve this spatial information, which might improve estimates.

In the 2D CNN, two convolutional layers surround an average pooling layer (Fig. 2b). Convolutions are performed over the “time” and “bin” dimensions while considering bands as channels. After the second convolutional layer, the data were fed into a spatial pyramid pooling layer (He et al., 2014). Besides the 3D histograms, this model also used categorical information about the provinces (in a one-hot encoded format) similarly to the 1D model. Therefore, one-hot encoded administrative codes were concatenated with the pooled features before being passed to the regression head. Finally, a simple dense layer with linear activation was applied to obtain the final prediction.

To control for overfitting in both networks, we used four regularization mechanisms: dropout with a tuned dropout rate (Table 2), a L2 regularization on the weights, i.e. weight-decay, applied for all the layers with a small rate of 10^−6^, a validation set corresponding to 5% of the training set, and batch normalization. The kernels were initialized with “He Normal” distribution (He et al., 2015) and “same” zero padding was used. Each convolutional layer was followed by the rectified linear unit activation function. The selected loss function was the mean squared error.

#### Hyperparameter optimization

The performance of CNN models depends largely on its hyperparameters. In general, optimization of hyperparameters aims at minimizing the generalization error (Bergstra & Bengio, 2012). We sampled the hyperparameter space described in Table 2 with tree-structured Parzen estimators, which are a form of Bayesian optimization (Bergstra et al., 2011).

While hyperparameter optimization helps minimize generalization error, it can be time consuming. To reduce computing time without sacrificing accuracy, we pruned unpromising trials, i.e. algorithm configurations which were unlikely to rank among those configurations delivering maximum accuracy. As a result, computing time focussed on trials with high potential. Pruning requires intermittent feedback to the optimizer so that it can compare the progress of the current trial with that of past trials and decide whether to terminate it early. However, conventional cross-validation does not accommodate intermittent feedback. One must wait for every fold to be evaluated to estimate accuracy. Here, we use the concept of greedy cross-validation which can operate with a pruning algorithm for early stopping.

#### Experiments

Experiments were conducted to determine both the hyperparameters and the accuracy of the model through cross-validation. Three independent datasets were required: the training set used to train the model, the validation set used to optimize the set of hyperparameters and the test set used to estimate the performance of the optimized model in prediction.

Cross-validation was designed bearing in mind the scope of the application, where yield forecasts are made for a year never seen by the model. Therefore, cross-validation folds were defined based on years (Meroni et al., 2021). First, *n* folds—one per year—were systematically defined for testing. A tercile split based on average yield was used to extract 6 validation years from the *n*-1 years. Data from one year of the validation years was held out at the validation time, and the remaining *n*-2 years were used for model training.

For each experiment, 100 combinations of hyperparameters were evaluated and unpromising trials could be terminated after a minimum of 6-folds. We retained the model that minimized the root mean square error (RMSE) between yield forecasts and observations in validation phase. The results of best-performing 1D and 2D CNN models were evaluated and compared with several ML and simple models which are briefly described in the following section.

### Benchmark models

We benchmark our 1D and 2D convolutional models to ML models, and two simple conventional models. In previous work, we developed a robust and automated ML pipeline to select the best features and model for yield prediction on a monthly basis between the start and the end of the growing season (Meroni et al., 2021). Five common machine learning regression algorithms were compared: gradient boosting (Friedman, 2001), least absolute shrinkage and selection operator (LASSO) (Tibshirani, 1996), random forest (Breiman, 2001), multi-layer perceptron (MLP) (Van Der Malsburg, 1986) and support vector regression with linear and radial basis function (SVR lin and SVR rbf) (Vapnik et al., 1996).

These ML models were benchmarked against two simple benchmark models, the null and the peak NDVI models. The yield prediction for a specific province-year under the naive null model was equal to the average yield for both before and after the year being considered. The peak NDVI model makes the assumption that yield is linearly associated with the seasonal maximum of NDVI at the administrative unit (*yield* = *a* max (NDVI) + *b*) (Becker-Reshef et al., 2010).

### Evaluation (performance) metrics

We used two performance metrics for evaluating the models: the root mean square error (RMSEp) and the relative RMSEp percentage (rRMSEp, obtained by normalizing the crop specific average yield). The metrics were aggregated at national level computing the average province-level error metric.

The models were evaluated on all of the eight forecasting months, from December to July. The best-performing set of hyperparameters was chosen based on average province-level rRMSEp. The workflow was written in Python, and the models were defined with Keras/TensorFlow libraries (Abadi et al., 2016; *Keras*, 2015/2022). It is a fully automated, end-to-end, processing tool (https://github.com/ec-jrc/ml4cast-yieldcnn) and it was executed on the JRC Big Data Platform (Soille et al., 2018) using a GPU node equipped with NVIDIA Quadro 8000.

## Results and discussion

**Fig. 4:**
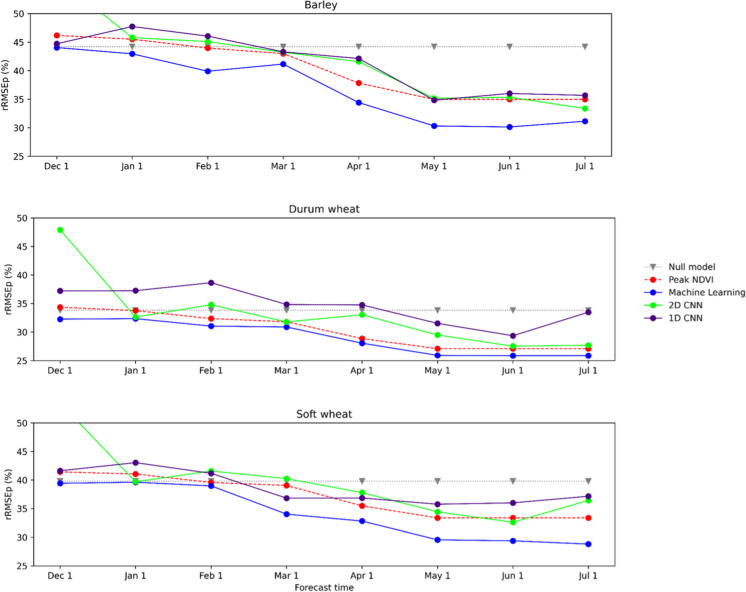
Relative root mean square error percentage (rRMSEp) for one- and two-dimensional convolutional neural network models, best-performing machine learning model, peak NDVI and null model

The best ML models, SVR, Lasso and MLP, consistently outperformed the benchmark models for each forecasting month. For the 1D CNN model, the results tend to improve after February forecast and become noticeably better after April (Fig. 4). The results deteriorate for durum wheat and last forecasting month. Soft and durum wheat yield predictions are unstable and tend to deteriorate towards the end of season, which is counter intuitive, i.e. inadequate learning. The 2D CNN model shows similar patterns as the 1D model with the yield forecasts stabilizing after February with slight disruptions in April for durum wheat. We conclude that 2D CNN forecasts were slightly better than 1D model for all crops, with the exception of the December forecast.

When comparing 1D CNN with the benchmark and ML, the results were never better than the best-performing ML model. The 1D CNN forecasts tend to improve after February; however, they are still not good enough to outperform the peak NDVI benchmark. ML forecasts were also consistently better than 2D CNN for all forecasting months. Peak NDVI also outperformed the 2D CNN model for almost all crops and forecasting months. 2D CNN provided slightly better forecast than the peak NDVI for July forecast for barley and for June forecast in the case of soft wheat.

**Fig. 5:**
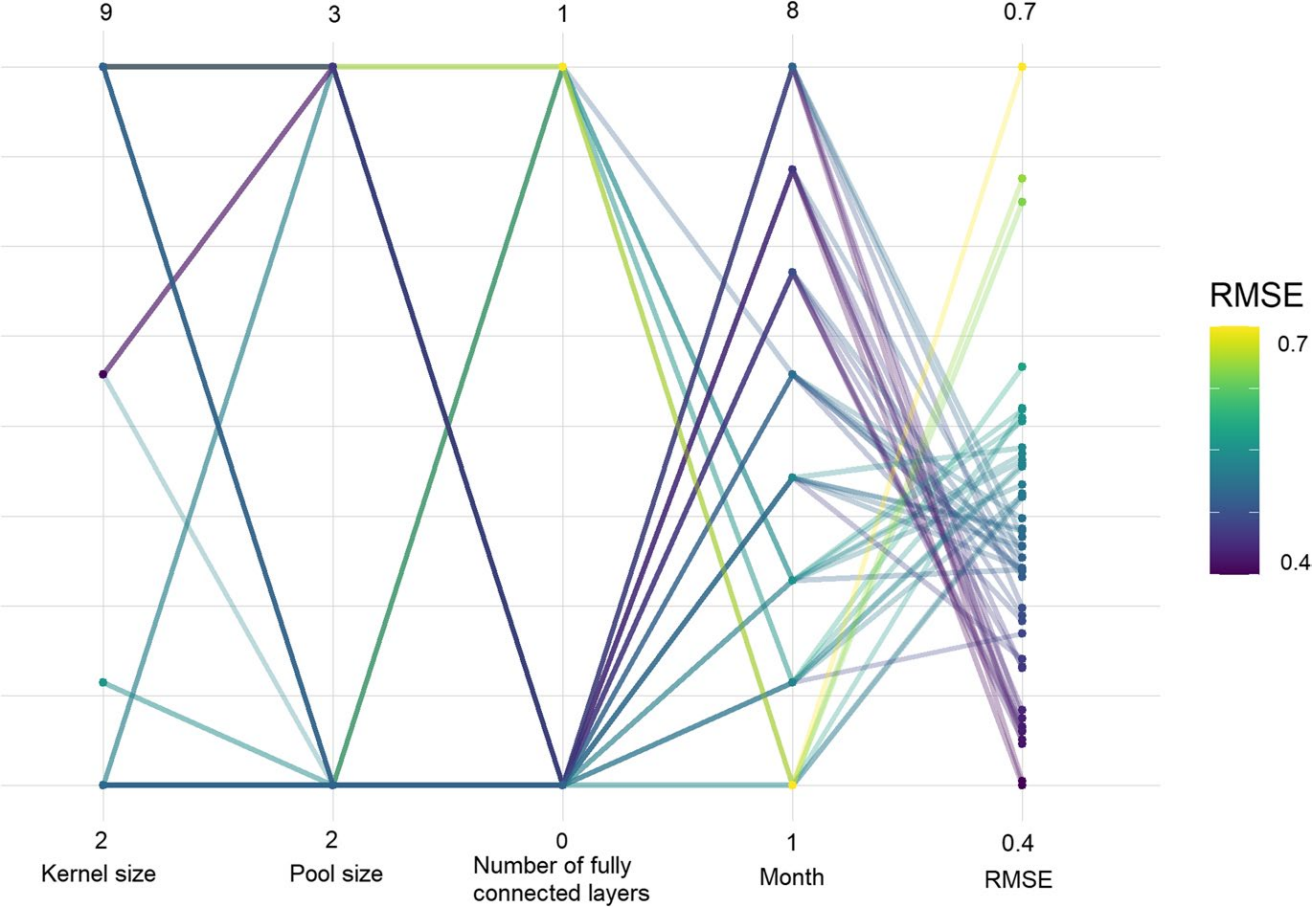
Selection of the best-performing hyperparameters for 1D and 2D models per forecasting month. Month 1 corresponds to December and Month 8 to July

Figure 5 shows an example of the best-performing 1D and 2D CNN hyperparameters selection per month (December (1) to July (8)) for all crops. Other hyperparameters were omitted due to clarity since they did not contribute to significantly higher or lower RMSE. This example demonstrated that the presence of dense or fully connected (FC) layers in the model contributed to higher RMSE values in prediction. The FC layers (0 or 1) tended to be selected for the first forecasting months in the case of 2D CNN which resulted in high RMSE values (Fig. 3). Most of 1D and 2D models were not using the FC layers which resulted in better yield predictions. Therefore, the regression head part of the model (Table 2) was not beneficial for yield forecasting in this case.

Even though there is a growing use of DL for crop yield forecasting and time series modelling, in this case study, we found no evidence that DL, given limited data, can perform better than ML and simple peak NDVI models. It is likely that the data set size hampers successful application of DL models despite the use of simple CNN architectures and that the number of parameters used for both models was adapted to the small amount of input data, 1600–84,000 parameters for 2D and 256–1800 parameters for 1D model, depending on the selected hyper parameters. Therefore, this case study suggests that the ML models still remain a better choice in a data limited context.

**Fig. 6:**
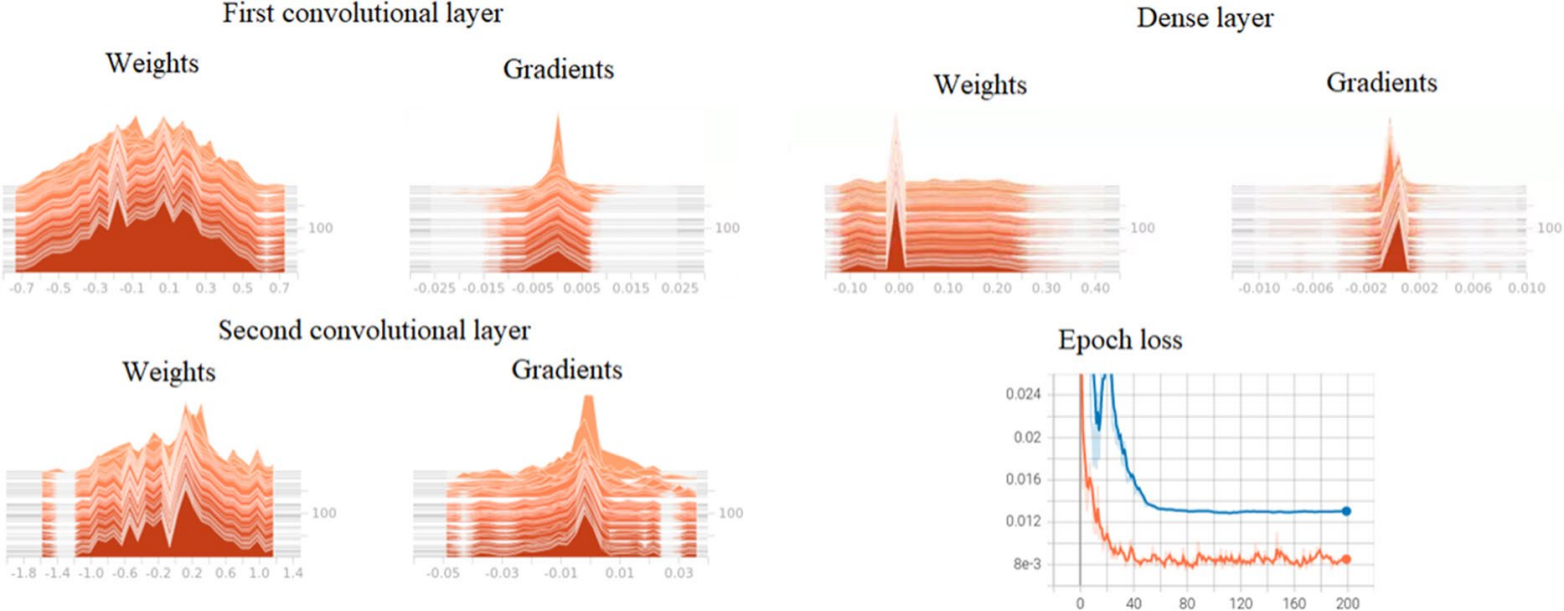
Histogram of kernel weights and gradients for the best-performing one-dimensional model. Validation and training losses are shown with blue and orange colours, respectively

To detect irregular network behaviour and explain the failure of the DL models training, we analysed the histograms of the network kernels and gradients. These histograms show the distribution of the kernel weights or gradient values over epochs. They can be useful for network debugging and detecting exploding (exponentially increasing derivatives) or vanishing gradients (exponentially decreasing derivatives). We show one example of these histograms in Fig. 6 for the best-performing 1D CNN model and one validation year. Most of the network activity occurred before epoch 40 when the training and validation loss stopped improving and the gradient updates remained constant as well as the kernel weights. There was no evidence of vanishing or exploding gradient as well as model overfitting.

Visual inspection of the kernel weights and gradients for other 1D (different validation years and crops) and 2D models showed very similar patterns throughout the epochs, with no signs of overfitting.

Transfer learning, an approach that has not been fully explored in the context of crop yield forecasting, may be used to improve the performances of DL models in data poor environments. In fact, due to their design, DL models can also be pre-trained in areas with abundant and good quality training data and be fine-tuned in another, data scarce, region. This approach requires fine-tuning of pre-trained models in order to be successfully applied for crop yield forecasting tasks (Khaki et al., 2021; Wang et al., 2018) Pre-training a network on similar neighbouring countries will be investigated in a future study.

## Conclusion

We developed a fully automated deep learning workflow to forecast crop yields with publicly available climate and satellite time series data. Two types of DL models, 1D and 2D CNNs with flexible configurations, were developed and an extensive hyperparameter tuning procedure was applied in order to select the best model configuration for each crop and each forecast month. The best-performing DL models were then compared with ML and benchmark models. Both workflows were applied in Algeria, using the same input data, to predict barley, soft and durum wheat regional yields on a monthly basis for a period 2002–2018. There was no significant added value for forecasts made with DL compared to the best-performing ML models and peak NDVI model. These results contribute to an understanding of how DL models can perform in a limited data context as compared to ML and simple benchmark models.

## Data Availability

Python codes, for ML and DL models, are available on the JRC GitHub repository: https://github.com/ec-jrc/ml4cast-ml; https://github.com/ec-jrc/ml4cast-yieldcnn.
